# Lipoprotein Lipase, Tissue Expression and Effects on Genes Related to Fatty Acid Synthesis in Goat Mammary Epithelial Cells

**DOI:** 10.3390/ijms151222757

**Published:** 2014-12-09

**Authors:** Wang-Sheng Zhao, Shi-Liang Hu, Kang Yu, Hui Wang, Wei Wang, Juan Loor, Jun Luo

**Affiliations:** 1 Shaanxi Key Laboratory of Molecular Biology for Agriculture, College of Animal Science and Technology, Northwest A&F University, Yangling 712100, China; E-Mails: genetics@nwsuaf.edu.cn (W.-S.Z.); shilq.hu@gmail.com (S.-L.H.); yk20050817@gmail.com (K.Y.); huitomorrow520@gmail.com (H.W.); wangweixn007@nwsuaf.edu.cn (W.W.); 2 Mammalian NutriPhysioGenomics, Department of Animal Sciences and Division of Nutritional Sciences, University of Illinois, Urbana, IL 61801, USA; E-Mail: jloor@illinois.edu

**Keywords:** *LPL* gene, lactation, goat mammary epithelial cells, Orlistat

## Abstract

Lipoprotein lipase (LPL) serves as a central factor in hydrolysis of triacylglycerol and uptake of free fatty acids from the plasma. However, there are limited data concerning the action of *LPL* on the regulation of milk fat synthesis in goat mammary gland. In this investigation, we describe the cloning and sequencing of the *LPL* gene from Xinong Saanen dairy goat mammary gland, along with a study of its phylogenetic relationships. Sequence analysis showed that goat *LPL* shares similarities with other species including sheep, bovine, human and mouse. *LPL* mRNA expression in various tissues determined by RT-qPCR revealed the highest expression in white adipose tissue, with lower expression in heart, lung, spleen, rumen, small intestine, mammary gland, and kidney. Expression was almost undetectable in liver and muscle. The expression profiles of *LPL* gene in mammary gland at early, peak, mid, late lactation, and the dry period were also measured. Compared with the dry period, *LPL* mRNA expression was markedly greater at early lactation. However, compared with early lactation, the expression was lower at peak lactation and mid lactation. Despite those differences, *LPL* mRNA expression was still greater at peak, mid, and late lactation compared with the dry period. Using goat mammary epithelial cells (GMEC), the *in vitro* knockdown of *LPL* via shRNA or with Orlistat resulted in a similar degree of down-regulation of *LPL* (respectively). Furthermore, knockdown of *LPL* was associated with reduced mRNA expression of *SREBF1*, *FASN*, *LIPE* and *PPARG* but greater expression of *FFAR3*. There was no effect on *ACACA* expression. Orlistat decreased expression of *LIPE*, *FASN*, *ACACA*, and *PPARG*, and increased *FFAR3* and *SREBF1* expression. The pattern of *LPL* expression was similar to the changes in milk fat percentage in lactating goats. Taken together, results suggest that *LPL* may play a crucial role in fatty acid synthesis.

## 1. Introduction

Lipoprotein lipase (*LPL*) (EC 3.1.1.34), initially defined as one of three members of the lipase gene family, is associated with the hydrolysis of the triacylglycerol (TAG) component of circulating chylomicrons and very low density lipoproteins (VLDL) causing the release of two non-esterified free fatty acids (NEFA) and a monoacylglycerol for tissue utilization [1,2].

The cDNA sequences of several mammalian *LPL* have been isolated and determined, including sheep, bovine, human, and mouse. The activity and mRNA expression of *LPL* has been investigated in a wide range of tissues including adipose tissue, heart, liver, skeletal muscle, lung, lactating mammary gland, brain, and kidney in mouse, rat and bovine [3,4]. The mRNA expression of *LPL* among various tissues depends on the physiological state of the animal, feeding/fasting, cold adaptation, nutrition, metabolic and transport activities [5,6,7,8,9].

Significant progress was made in understanding fatty acid synthesis within the mammary glands in the past decades. Kern *et al.* noted [10] that the milk fat was composed primarily of triglycerides and a small proportion of other lipids. Jensen indicated [5] 416 fatty acids existed in bovine milk lipids and there are dual sources of origin of milk fat. Fatty acids are either synthesized *de novo* of the short, medium-chain fatty acids in the mammary glands, or they are from dietary long-chain fatty acids (LCFA). *LPL* is generated in the epithelial cells of the mammary gland, and alters the release of fatty acids in mammary gland because *LPL* activity is higher in lactating mammary gland and epithelial cells are the prominent cell type remarkable [11,12]. These LCFA are imported from the plasma after being either released from triglycerides circulating in chylomicra or VLDL by the enzyme lipoprotein lipase (LPL) [13] or derived from the plasma NEFA.

Orlistat (tetrahydrolipstatin, THL), known as a potent, specific and irreversible inhibitor of lipoprotein lipase activity, was the first drug used to treat obesity [14,15]. Inhibition of *LPL* would prevent hydrolysis of dietary fat into absorbable NEFA, leading to the reduction of cholesterol and low-density lipoprotein [16]. To date, Orlistat has been the most commonly used inhibitor for exploring the enzymatic mechanism and possible function of *LPL*.

Obtaining knock-out goats for *in vivo* studies of *LPL* gene function related to milk fat synthesis in mammary gland during lactation is likely not feasible due to the high cost and concern for animal welfare. Furthermore, several papers describe the characteristics and regulations of *LPL* in mammary gland [17,18]. However, the regulatory mechanism of *LPL* gene in goat mammary epithelial cells and the correlation between mRNA expression of *LPL* gene and milk fat synthesis was still unclear. Our objectives were to obtain and characterize the full length cDNA of the *LPL* gene from the mammary gland of dairy goats, analyze the structure and construct phylogenetic tree, evaluate *LPL* mRNA expression in 10 tissues and at five stages of lactation, and lastly study the potential effect of *LPL* on milk fat synthesis genes in goat mammary epithelial cells (GMEC).

## 2. Results and Discussion

### 2.1. Cloning and Sequence Analysis of Lipoprotein Lipase (LPL)

The full-length *LPL* cDNA consists of 3555 nucleotides, including 142 bp of 5' UTR, 1437 bp of the ORF and 1976 bp of 3' UTR, which is capable of encoding a polypeptide of 478 amino acids with an estimated molecular mass of 53.38 kDa and a predicted isoelectric point (pI) of 8.72. The sequence of *LPL* contains an apparent polyadenylation signal with a poly (A) tail. The goat *LPL* cDNA sequence was submitted to the GenBank database (GenBank accession Number: JQ670882).

The results of the BLAST (blastp) search on the NCBI website according to the inferred amino acid sequence of *LPL* indicated greater similarity between goat and sheep, compared with bovine, pig, human, and mouse. The 9 bp representing three amino acids between 88 and 102 nt of coding region is absent in human and mouse, indicating variations of the potential motif site. The nucleotide homology among Xuhuai goat (NM_001285607), sheep (NM_001009394), bovine (NM_001075120), pig (AY559453), human (NM_000237) and mouse (NM_008509) were 99%, 98%, 98%, 91%, 89% and 87%, respectively. The homology of amino acid sequences was 100%, 98%, 98%, 92%, 90% and 88%, respectively. However, the 5' UTR domain differs among these species.

The presence of trans-membrane regions within *LPL* protein was predicted using the online software TMHMM, however, results indicated the absence of trans-membrane structure (Figure S1). A signal peptide search using SignalP software (CBS, Kemitorvet, Denmark) identified the signal peptide cleavage site between 23–24 amino acids, which is consistent with the position reported in Xuhuai goat [17], sheep and bovine (data not shown). The probability of a signalpeptide cleavage site in our study was 91.8%, which was higher than that of the Xuhuai goat (up to 65.9%) [17] and could be related to breed differences. There exist four poly-A (AATAAA) signals in the 3' UTR of the Xinong Saanen dairy goat *LPL*, suggesting that there may be multiple polyadenylation sites responsible for post-transcriptional modifications.

### 2.2. Phylogenetic Analysis of LPL Protein

**Figure 1:**
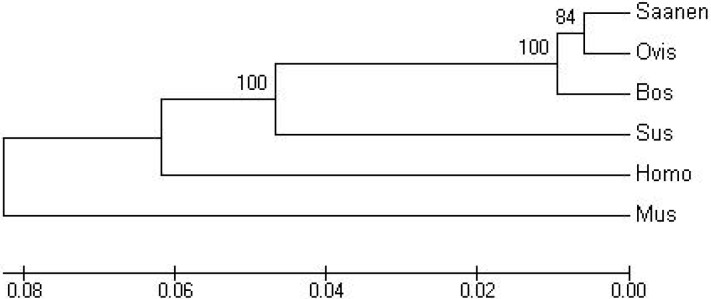
Neighbor-joining (NJ) phylogeny describing the similarity of CDS sequence of goat (Capra) *LPL* gene with the sequences of sheep (Ovis), bovine (Bos), pig (Sus), human (Homo), and mouse (Mus).

A phylogenetic tree was constructed and analyzed based on the deduced amino acid sequence of *LPL* from six species. The genetic distance among these six species was determined using the neighbor-joining (NJ) method [19]. A comparison of the distance among these six species revealed that *LPL* from goat and sheep grouped into one cluster with another moderately related cluster grouping bovine and swine, suggesting that *LPL* is more conserved between goat and sheep compared with the other four species. The most distant cluster from goat and sheep was that of human and rat. The distances among various species further verified the evolutionary relationship of these proteins (Figure 1). In addition, the order of branching for mammals represented in the *LPL* phylogeny is in accordance with the previous results suggested by Li *et al.* [20], in which rodents were one of the earliest groups to separate off from other mammals.

### 2.3. The Expression Profiles of LPL in 10 Tissues and Different Stages of Lactation

**Figure 2:**
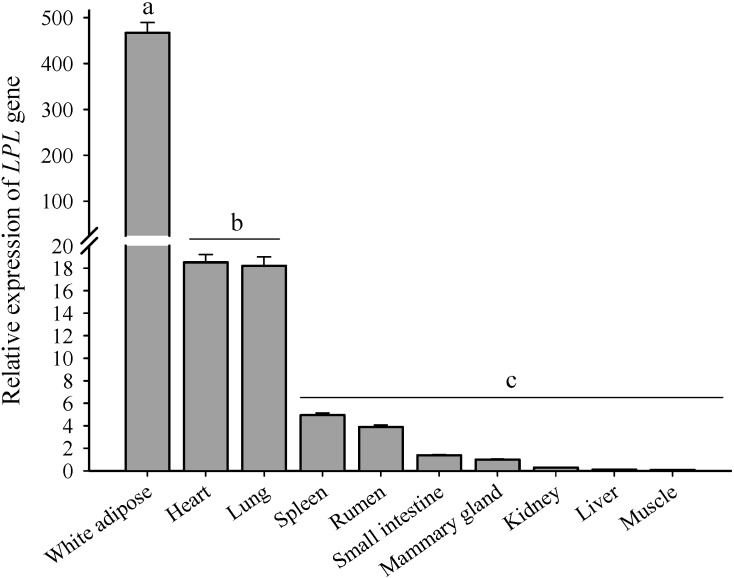
Relative expression of *LPL* in 10 different tissues from goat. RNA examples and RT-qPCR was performed in triplicate. The results were expressed relative to the mammary gland (named 1). Data are calculated using mean ± SD. Dissimilar letters (a, b, and c) denote significant differences between different tissues (*p* < 0.05).

Expression of *LPL* mRNA from 10 tissues including mammary gland, white adipose tissue, heart, lung, rumen, kidney, small intestine, spleen, muscle and liver was measured by RT-qPCR (Figure 2). The results showed that *LPL* is expressed in almost all tissues, albeit at very different levels, with the greatest expression in the white adipose tissue. In addition, expression of *LPL* was relatively higher in heart and lung. Relative expression of *LPL* was lower than other tissues and similar among spleen, rumen, small intestine, mammary gland and kidney. The expression of *LPL* in liver and muscle was undetectable. A similar pattern across all the tissues was also seen by other investigators [21,22].

In agreement with previously reported results in guinea pig [21] and bovine [22], mRNA expression of *LPL* gene was highest in adipose tissue, heart and lung which suggests that the highest *LPL* expression in these three tissues is likely due to the greater utilization of fatty acids as energy sources or building blocks for TG. The very low expression detected in the spleen, rumen, small intestine, kidney and liver suggests that *LPL* in these tissues might not be necessary as these tissues do not readily utilize circulating fatty acids [13,23]. Nevertheless, *LPL* may be involved in specialized functions (*i.e.*, surfactant production, tissue repair and whole-body energy homeostasis). The expression of *LPL* in mammary gland and muscle compared with other tissues was slightly lower than reported in other species. Furthermore, the presence of *LPL* mRNA level was 5–15-fold lower in goat muscles and mammary gland compared to that in these two tissues of rat and bovine, which has been previously reported [5,22,24]. Such differences may be due to nutritional status and also different protocols for gene expression analysis used.

**Figure 3:**
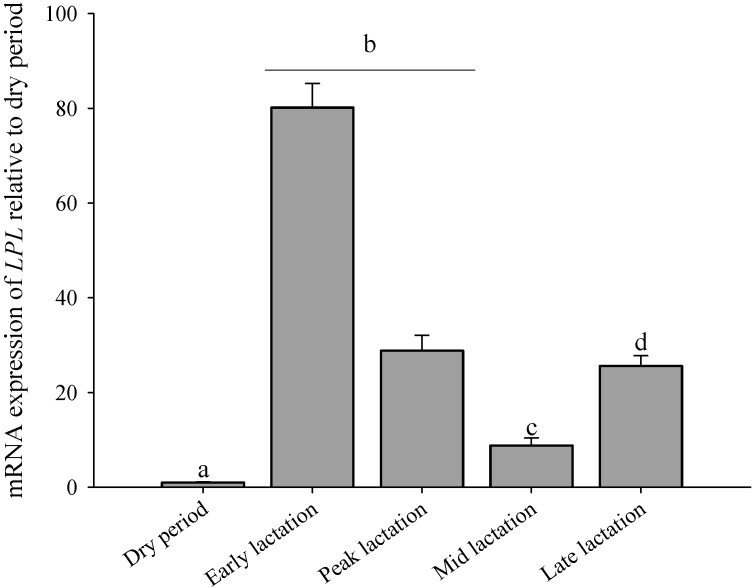
Relative expression analysis of *LPL* gene in goat mammary gland among five different stages of lactation. The results were expressed relative to the dry period (named 1). Data are calculated using mean ± SD. Dissimilar letters (a, b, c, and d) denote significant differences between different lactations (*p* < 0.05).

The expression profiles of *LPL* in different lactation stages of mammary gland revealed a dramatic increase between the dry period and onset of lactation, reaching maximum levels within about one month at early lactation. A very rapid decline in *LPL* mRNA followed at peak lactation and mid lactation compared with early lactation. More than twofold increase from mid lactation to late lactation was statistically significant and that response was followed by nearly undetectable expression during the dry period (Figure 3).

During the transition from late pregnancy to lactation, the goat mammary gland experiences a marked up-regulation of genes (e.g., *LPL*) involved in lipid metabolism and nutrient transport to support the high levels of milk production [25], which is in accordance with the findings in mammary gland of bovine [6,18] mouse [26], and human [27]. In early lactation, the goats used in the present study were in negative energy balance leading to mobilization of fatty acids from adipose tissue towards different tissues including liver and mammary gland to meet energy requirements. Since FFA obtained through the hydrolysis of circulating TAG by mammary *LPL* represent a significant source of milk fatty acids [28], the greater *LPL* mRNA expression in goat mammary gland suggests that it could have enhanced the hydrolysis of TAG packaged into the VLDL fraction secreted by the liver.

Earlier research in goats reported a high concentration of plasma prolactin preceding the initial extraction of TAG from circulation by the mammary gland (MG) [29] which agrees with our observations of a significant increase in *LPL* during early lactation that remained elevated throughout lactation. It was hypothesized that the up-regulation of *LPL* in mammary gland is mediated by the anterior pituitary via the release of prolactin [29], which agrees with the concept of hormonal regulation of mammary adaptations to lactation [30]. Changes in *LPL* gene expression during lactation followed the same trend as the level of free fatty acids (FFA) likely is partly responsible for the process of lipolysis in milk.

### 2.4. Effects of LPL Knockdown and Orlistat on mRNA Expression of Genes Related to Milk Fat Synthesis in Goat Mammary Epithelial Cells (GMEC)

**Figure 4:**
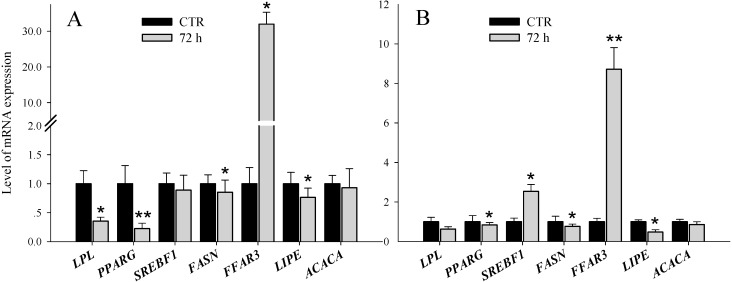
The mRNA expression of genes involved in milk fat synthesis after *LPL* knockdown and adding Orlistat in goat mammary epithelial cells. (**A**) The expression of genes related to milk fat synthesis after *LPL* knockdown in goat mammary epithelial cells (GMEC); and (**B**) The expression of genes related to milk fat synthesis after adding Orlistat in GMEC. All the genes were evaluated using RT-qPCR in GMEC after treatments for 72 h. Data are calculated using mean ± SD. All samples were prepared in triplicate. * *p* < 0.05 *vs.* control group, ** *p* < 0.01 *vs.* control group.

Expression of *LPL* was significantly lower with both *LPL* knockdown and addition of Orlistat in GMEC. Compared with the control, knockdown of *LPL* reduced expression of *SREBF1*, *FASN*, *LIPE* and *PPARG* by 11%, 15%, 24% and 78%, respectively, and increased the expression of *FFAR3* by 3199%. No effect was observed for *ACACA* (Figure 4A). Similar results were observed with addition of Orlistat, *i.e.*, the expression of *LIPE* (52%) was dramatically inhibited and *FFAR3* (872%) was induced sharply together with as down-regulation of *FASN* (25%) and *PPARG* (16%) and without effect on *ACACA*. The marked up-regulation of *SREBF1* (254%) was unexpected (Figure 4B).

Relative to the control, the expression of free fatty acid receptor 3 (*FFAR3*) was sharply up-regulated (3199% and 872%), which was very surprising. Current evidence suggests that *LPL* serves as a vital factor in regulating the supply of fatty acids obtained through the hydrolysis of circulating TAG in mammary glands [6,28]. Hansen and colleagues [31,32,33] discussed aspects of TAG synthesis and the specific role of the short chain and medium-chain fatty acids in TAG synthesis in goat mammary glands. They underscored the existence of a balance between the rate of endogenously-activated fatty acids in the mammary gland, rate of *de novo* synthesis, and the supply of alpha-glycerol phosphate for milk fat synthesis. Therefore, it is tempting to speculate that with the inhibition of mRNA expression of *LPL*, the supply of preformed fatty acids for esterification into triacylglycerol within GMEC decreased. Accordingly, the relative concentration of short-chain and medium-chain fatty acids may have increased to maintain the balance of fatty acids in GMEC. *FFAR3* has been recently cloned in bovine mammary epithelial cells (bMEC) and was reported to bind with short-chain fatty acids (SCFAs) [34]. Thus, with the possible increase of SCFAs in GMEC via *LPL* inhibition, it was supposed that the expression of *FFAR3* could be induced accordingly.

It was noteworthy that *PPARG* expression decreased after *LPL* knockdown in GMEC. The promoter region of *LPL* contains a *PPARG* response element [35,36]. A previous study discussed existence of a positive regulatory feedback loop between *PPARG* and *LPL* [36]. With the decrease of *LPL*, the mRNA expression of *PPARG* would decrease concurrently. A similar mechanism of regulation also has been proposed in adipose tissue [37]. Data suggest that *PPARG* may control the expression of genes involved in TAG hydrolysis in GMEC [38]. Because *PPARG* serves as the main regulator of TAG synthesis and secretion, we propose that the dramatic reduction of *LPL* expression in GMEC decreases the need to incorporate preformed fatty acids into triacylglycerol and therefore the expression of *PPARG* should decrease.

As a chemical inhibitor of *LPL*, it has been shown that Orlistat can decrease the release of NEFA (free fatty acid, FFA) into cell culture media [39]. In addition, several types of LCFAs (e.g., palmitic acid) appeared to act as the activating ligands for *PPARG* [9,40]. The decrease of LCFAs released into the medium due to lower *LPL* expression would also result in suppression of *PPARG* expression.

An issue of concern is whether downstream regulation of *LPL*-derived fatty acids is as important in the mammary gland as it is in adipose tissue. *LIPE* (Lipase, hormone-sensitive) is the enzyme participating in mobilization of intracellular TAG in adipose tissue; it is regulated post-translationally being activated by phosphorylation and suppressed by dephosphorylation [41]. It appears that a coordinated regulation of *LPL*, *LIPE* and fatty acid esterification is responsible for fatty acid mobilization and storage in adipose tissue [42]. However, whether a similar mechanism between *LPL* and *LIPE* exists in the MG needs to be determined. Moreover, the reduction of *LPL* alone was not sufficient to explain the down-regulation of *LIPE*. The action of *LIPE* is involved in the release of FFAs from TAG and may be related to the increasing expression of *LPL* in epithelial cells [43].

In mammals, numerous genes involved in milk fat synthesis, including *FASN* [44], *ACACA* [45], and *LPL* [35,46], are all known target genes of *SREBF1* [35,47,48]. Our data suggest that goat *ACACA* is not regulated by up-regulation of *SREBF1*. Alternatively, there also may be indirect effects such that the change of LCFA in medium via the decrease of *LPL* expression may alter lipogenic gene expression (e.g., binding to other *PPAR* or nuclear receptors) on *SREBF1* mediated mechanisms [49]. Together, these data indicate that the molecular mechanisms of *LPL* inhibition acting indirectly on *ACACA* and *FASN* via *SREBF1* still need to be investigated.

The response of *SREBF1* to Orlistat was markedly different compared with the knockdown of *LPL*. The reason for this inconsistency is unclear, but could be taken as indication of different mechanisms between Orlistat and knockdown of *LPL*. Additionally, whether there was a direct effect of Orlistat on *SREBF1* still needs to be determined.

Overall, our data revealed that the expression of *LPL* in various tissues of goats has a similar profile as in bovine [22]; however, there are some variations likely due to differences in developmental regulation within tissues. The actions of RNAi and Orlistat on *LPL* in GMEC still need to be explored in future work.

## 3. Experimental Section

### 3.1. Animals and Samples Collection

Six Xinong Saanen dairy goats (4 years old, third parity) were selected and divided into two groups of three to conduct the main project (Experimental farm of the Northwest A&F University, Yangling, Shaanxi, China). For one group, all three goats were slaughtered during dry period (30 day) and the tissue samples of heart, kidney, liver, lung, mammary gland, muscle, rumen, small intestine, spleen and white adipose tissue were collected, washed with phosphate-buffered saline (PBS) and instantly frozen in liquid nitrogen. For the other group, mammary gland biopsies were conducted from alternate glands in each goat under sterile procedures at five time points: Early lactation (28 day), peak lactation (60 day), mid-lactation (100 day), late lactation (270 day), and dry period (60 day prior to parturition). The mammary gland (MG) biopsies were conducted after milking with no feed provided on the morning of the surgery. The mammary gland samples from lactating goats were obtained via surgical procedure as described by Farr *et al* [50]. Briefly, a midpoint (about 10 cm^2^ area of skin) on the right or left rear quarter gland was selected as surgical site. The biopsy areas were carefully selected to ensure the area was free from scar tissue from any previous biopsies. Before the injection of general anesthesia, the site for biopsy on the mammary gland of the goats was shaved and cleaned. A 3–4 cm incision was made through the skin using a scalpel to avoid any large subcutaneous blood vessels, and then blunt dissection of the mammary capsule was performed to ensure tissue obtained was mammary parenchyma during the biopsy. About 2–3 g of MG tissue was collected and frozen in liquid nitrogen instantly and stored at −80 °C prior to RNA extraction. The incision was closed with sutures and Acramide powder was applied. All sutures were removed approximately 30 day after surgery. All animal procedures and experiments were conducted based on the guidelines approved by the Committee (20080926, on 6 October 2008) for Animal Care and Use at Northwest A&F University, China.

### 3.2. Cloning, Sequencing Analysis and Tissue Expression of LPL

#### 3.2.1. Total RNA Extraction, cDNA Synthesis and Quantification

TRIzol reagent (Invitrogen, Carlsbad, CA, USA) was used to isolate and purify RNA from each tissue sample following the manufacturer’s instructions. RNA concentration was determined by a NanoDrop ND-2000 spectrophotometer (NanoDrop Technologies, Wilmington, DE, USA) and the integrity of RNA were verified via agarose gel electrophoresis of the 28S and 18S rRNA subunits. Meanwhile, extracted RNA from all tissues was diluted to a consistent concentration (100 ng/μL) for gene expression analysis. The extracted total RNA from mammary gland of three slaughtered goats was divided into two groups: One was used for cloning cDNA of *LPL* gene, the second one was used for processing Real Time quantitative PCR (RT-qPCR) together with RNA samples extracted from another nine various tissues. Another RNA samples were extracted and prepared from mammary gland biopsies of the other three goats for running RT-qPCR in various lactation stages. The first strand cDNA of different tissues were synthesized by reverse transcription PCR using the PrimeScript^®^ RT kit (Takara Biotechnology Co., Ltd., Dalian, China).

The qPCR was performed using 8 μL diluted cDNA combined with 12 μL of a mixture. The mixture was composed by 10 μL 2XSYBR Green Master Mix (Takara Biotechnology Co., Ltd.), 0.8 μL each of 10 μM forward and reverse primers, and 0.4 μL RNase free water. *GADPH*, *UXT*, and *RPS9* were used as the reference genes and the details on selected lipid metabolism genes were listed in Table S1. *GADPH*, *UXT*, and *RPS9* were selected because *GAPDH* has been used previously in a goat mammary tissue study [51], and also because *UXT*, and *RPS9* were the most stably-expressed genes and have been verified as suitable internal control genes in bovine mammary studies [52]. Methods for primers design and validation were reported as previously [18].

Each sample and a 6-point relative standard curve plus the non-template control were run in triplicate. Total of 500 ng RNA in the reaction was used and all qPCR reactions were performed on Applied Biosystems 7300 Real-Time PCR System (Applied Biosystems, Inc., Foster City, CA, USA) at 95 °C for 10 min, followed by 40 cycles at 95 °C for 15 s, and 60 °C for 1 min. The presence of a single PCR product was verified by the dissociation protocol using incremental temperatures to 95 °C for 15 s, 60 °C for 1 min, 95 °C for 15 s and 60 °C for 15 s.

#### 3.2.2. Full-Length Cloning of *LPL* cDNA

As shown in Table S2, by using the released gene sequence of bovine (GenBank ID: NM_001075120) and human (GenBank ID: NM_000237) in the National Centre for Biotechnology Information (NCBI) database and conducting similar alignment of the conserved domains, all the primers were designed and synthesized by Invitrogen. PCR was performed using the primers *LPLF* and *LPLR* based on three steps, with 95 °C 5 min for denaturation (1 cycle), followed by 94 °C 30 s, 57 °C 30 s, and 72 °C 90 s (32 cycles), then 72 °C 10 min for extension (1 cycle) and, finally, at 4 °C. The cloning of cDNA ends (5' and 3' UTR) were achieved using 5' RACE System for Rapid Amplification of cDNA Ends, Version 2.0 (Invitrogen, Carlsbad, CA, USA) and 3'-Full RACE Core Set Ver.2.0 (Takara Biotechnology Co., Ltd., Dalian, China) by following the manufacturer’s manuals. The specific primers for 5' (*LPL*-5R, see Table S1) and 3' RACE (*LPL*-3F1 and *LPL*-3F2, and *LPL*-3R, see Table S1) were designed according to the ORF sequence, respectively. All PCR products were cloned into pGEM-T Easy vectors (Promega, Beijing, China), and then were sent to sequence in Invitrogen Life Technologies™ (Invitrogen, Carlsbad, CA, USA).

#### 3.2.3. Bioinformatics Analysis of *LPL*

The ORF and matching amino acid, plus protein molecular weight (MW) and isoelectric point (pI) value were determined using Bio-XM software (version 2.6) (H. S. Zhang, Nanjing, China) (http://zhanglab.njau.edu.cn/bioxmsetup26.exe). The similarities of nucleotide and assumptive amino acid sequences among diverse species were performed using BLAST search at NCBI (http://blast.ncbi.nlm.nih.gov/Blast.cgi). The prediction of trans-membrane structure and signal peptide sequence was analyzed using the online bioinformatics tools (http://www.cbs.dtu.dk/services/TMHMM/) and (http://www.cbs.dtu.dk/services/SignalP/). The phylogenetic tree was generated using the ClustalW2 (EMBL, Hinxton, United Kingdom) (http://www.ebi.ac.uk/Tools/phylogeny/clustalw2_phylogeny/) and MEGA 4 programs (S. Kumar, Tempe, AZ, USA) (http://www.megasoftware.net/mega4/mega.html).

### 3.3. Expression and Statistical Analysis

The amplification efficiency for each gene was calculated using the standard curve method (*E* = 10^−1/(−log curveslope)^) as reported previously [18]. Relative expression of *LPL* gene was measured and analyzed by applying comparative *C*_t_ (ΔΔ*C*_t_) method [53]. Δ*C*_t_ = *C*_t_ sample − geometric mean *C*_t_ of 3 internal control genes, The RT-qPCR results were normalized and expressed as mean ± standard deviation (SD). The statistical significance for various tissues expression of *LPL*, mRNA expression of *LPL* in different lactation stages and effects of RNA interference and Orlistat on milk fat synthesis genes were all determined by using one-way ANOVA with Duncan’s *post hoc* tests. All statistical computations were conducted using SPSS 18.0 software (IBM, Armonk, NY, USA). *p* < 0.05 was set as the significance level.

### 3.4. Effects of Orlistat (Tetrahydrolipstain, THL) and LPL Knockdown on mRNA Expression of LPL and Genes Related to Milk Fat Synthesis in GMEC

#### 3.4.1. Adenovirus Establishment

To inhibit *LPL* in GMEC, shRNA expression cassette was first constructed to pENTR vector including an EGFP reporter gene and then were shifted into an adenoviral vector (pAd/PL-DEST) using the Gateway method (Invitrogen) to generate pAd shRNAvectors. The sequences of shRNA-429 and shRNA-NC pairs were as follows:

shRNA-429-sense: 5'-GATCCGGATGGCGGATGAATTTAAGAGTACTGTTAAATTCATCCGCCATCCTTTTTTC-3' (Bam H I restriction site underlined).

shRNA-429-antisense: 5'-TCGAGAAAAAAGGATGGCGGATGAATTTAAcAGTACTCTTAAATTCATCCGCCATCCG-3' (Xho I restriction site underlined).

shRNA-NC-sense: 5'-GATCCACTACCGTTGTTATAGGTGGAGTACTGCACCTATAACAACGGTAGTTTTTTTC-3' (Bam H I restriction site underlined).

shRNA-NC-antisense: 5'-TCGAGAAAAAAACTACCGTTGTTATAGGTGCAGTACTCCACCTATAACAACGGTAGTG-3' (Xho I restriction site underlined).

*Pac I* linearized adenoviral plasmids pAd-shRNA vectors were transfected into 293A cells using lipofectamine^2000^ (Invitrogen, Carlsbad, CA, USA). Ten days after transfection, the medium containing adenovirus was collected and subjected to two rounds of propagation in 293A cells following the previous protocol [54]. When the confluence of GMEC reached 70%–80%, adenovirus supernatant at a multiplicity of infection (MOI) of 200 was used to infect the cells. The medium was replaced with fresh medium 6 h later. The shRNA negative control adenovirus (Ad-NC) was used as a control. Cells were harvested 72 h after infection. All the samples were prepared in triplicate for RNA extraction and RT-qPCR.

#### 3.4.2. Cell Culture and Treatments

GMEC cells were cultured as previously reported [38,55] and maintained in the same conditions of 5% CO_2_ in air at 37 °C using the same medium with exception of insulin to prevent undesired effects on *LPL* gene. Cells were rinsed twice with PBS without Ca and Mg, 0.25% trypsin and serum-free DMEM/F-12 without antibiotics before initializing the experiment. Cells were cultured in a medium with D-MEM/F-12, hydrocortisone, EGF, penicillin/streptomycin, plus 10% FBS including 1 μM Orlistat (Roche Pharmaceuticals, Nutley, NJ, USA) until the confluence of cells was approximately 90%. Orlistat was dissolved in 100% ethanol and triplicate cultures of GMEC were cultivated for 72 h with this specific *LPL* inhibitor.

## 4. Conclusions

In summary, this study suggested that *LPL* gene contributed a potential role in TAG synthesis during lactation in goat mammary gland. The absence of trans-membrane structure and a signal peptide between 23–24 amino acid has been predicted using bioinformatics method. Moreover, the expression profiles of *LPL* gene in various goat tissues and different stages of lactation were also evaluated as a reflection of its role on mammary gland. A positive correlation between mRNA expression of *LPL* gene and milk fat synthesis was observed in different lactation stages of goats, which suggests *LPL* could be regulated by milk FA secretion in mammary gland. Inhibition of *LPL* by RNA interference and Orlistat in GMEC has implied that lipogenic gene expression within GMEC can be regulated by the circulating VLDL, chylomicrons and exogenous fatty acid through the potential effect of *LPL* indirectly. Further studies needs to be performed in order to elucidate the mechanisms underlying these regulatory processes.

## Supplementary Materials

Supplementary materials can be accessed at: http://www.mdpi.com/1422-0067/15/12/22757/s1.

## Abbreviation

*ACACA*: acetyl-coenzyme A carboxylase alpha
ANOVA: analysis of variance
bp: base pair(s)
BLAST: basic local alignment search tool
bMEC: bovine mammary epithelial cells
cDNA: DNA complimentary to RNA
CDS: coding sequence
CTR: control
DMEM: Dulbecco’s modified Eagle medium
EC: enzyme class
EGF: epidermal growth factor
FASN: fatty acid synthase
FBS: fetal bovine serum
FFA: free fatty acids
*GAPDH*: glyceraldehyde-3-phosphate dehydrogenase
GMEC: goat mammary epithelial cells
*FFAR3*: free fatty acid receptor 3
*LIPE*: lipase, hormone-sensitive
kDa: kilo Dalton(s)
LCFA: long chain fatty acid
*LPL*: Lipoprotein lipase
MG: mammary gland
MOI: multiplicity of infection
MW: molecular weight
NC: negative control
NCBI: national center for biotechnology information
NEFA: non-esterified fatty acids
NJ: neighbor-joining
nt: nucleotide
ORF: open reading frame
PBS: phosphate buffered saline
pI: isoelectric point
*PPARG*: peroxisome proliferator-activated receptor-γ
RACE: rapid amplification of cDNA ends
*RPS9*: ribosomal protein S9
RT-PCR: reverse transcription polymerase chain reaction
SCFAs: short-chain fatty acids
SD: standard deviation
shRNA: short hairpin RNA
*SREBF1*: sterol regulatory element binding factor1
TAG: triacylgleryerol
TG: triglyceride
UTR: un-translated region(s)
*UXT*: Ubiquitously expressed transcript protein
VLDL: very-low density lipoproteins
